# Digital Twin Approach for Fault Diagnosis in Photovoltaic Plant DC–DC Converters

**DOI:** 10.3390/s25144323

**Published:** 2025-07-10

**Authors:** Pablo José Hueros-Barrios, Francisco Javier Rodríguez Sánchez, Pedro Martín Sánchez, Carlos Santos-Pérez, Ariya Sangwongwanich, Mateja Novak, Frede Blaabjerg

**Affiliations:** 1 Department of Electronics, University of Alcalá, 28801 Madrid, Spain; franciscoj.rodriguez@uah.es (F.J.R.S.); pedro.martin@uah.es (P.M.S.); 2 Department of Signal Theory and Communications, University of Alcalá, 28801 Madrid, Spain; carlos.santos@uah.es; 3 AAU Energy, Aalborg University, 9220 Aalborg, Denmark; ars@energy.aau.dk (A.S.); fbl@energy.aau.dk (F.B.)

**Keywords:** digital twin, photovoltaic, fault diagnosis, machine learning, smart sensors, converters

## Abstract

This article presents a hybrid fault diagnosis framework for DC–DC converters in photovoltaic (PV) systems, combining digital twin (DT) modelling and detection with machine learning anomaly classification. The proposed method addresses both hardware faults such as open and short circuits in insulated-gate bipolar transistors (IGBTs) and diodes and sensor-level false data injection attacks (FDIAs). A five-dimensional DT architecture is employed, where a virtual entity implemented using FMI-compliant FMUs interacts with a real-time emulated physical plant. Fault detection is performed by comparing the real-time system behaviour with DT predictions, using dynamic thresholds based on power, voltage, and current sensors errors. Once a discrepancy is flagged, a second step classifier processes normalized time-series windows to identify the specific fault type. Synthetic training data are generated using emulation models under normal and faulty conditions, and feature vectors are constructed using a compact, interpretable set of statistical and spectral descriptors. The model was validated using OPAL-RT Hardware in the Loop emulations. The results show high classification accuracy, robustness to environmental fluctuations, and transferability across system configurations. The framework also demonstrates compatibility with low-cost deployment hardware, confirming its practical applicability for fault diagnosis in real-world PV systems.

## 1. Introduction

There is currently a significant increase in the deployment of renewable energy technologies, particularly solar PV systems. Additionally, the emergence of new paradigms, such as the rise of prosumers, continues to accelerate the integration of PV systems into the distribution grid. As a result, power converters have become a critical component in renewable energy systems, requiring high reliability to ensure stable and efficient operation. To address this need, several strategies have been explored, including fault-tolerant topologies with redundant components [1] and the development of highly reliable power electronic devices utilizing advanced materials [2]. Nevertheless, despite advancements in these areas, power converter components remain susceptible to faults. This has led to increased research into condition monitoring systems aimed at detecting potential failures in converter components [3]. Several studies have also analysed the reliability of PV plants in real-world conditions [4,5]. In [4], the authors conducted a reliability analysis of the hardware components in a PV plant, including PV modules, converters, string protection systems, and disconnection switches. Their findings indicate that power converters are the most critical components in terms of reliability, followed by string protections, PV modules, and AC/DC disconnection switches. Similarly, in [5], real operational data from 63 PV plants over a five-year period were analysed. The study found that the most frequently failing components were those associated with the PV modules, such as switches, blocking diodes, and fuses, followed by power converters. However, both studies ultimately converge on the same conclusion: power converters are the most critical and failure-prone components in PV systems. Therefore, special attention must be given to improving the reliability of converter components to ensure the long-term performance and availability of PV power plants. One of the emerging advancements being adopted across different fields is the use of a DT, a virtual model running in parallel with the real system [6,7]. The concept of the “Digital Twin” was first introduced by Grieves in 2003 [8], comprising three core components: the physical entity, the virtual entity, and the information exchange between them. In this work, a DT architecture is adopted that enables continuous real-time comparison between the virtual and physical systems. This comparison allows for accurate assessment of the system’s current state, early detection of anomalies, and their subsequent classification.

## 2. State-of-the-Art

In recent years, different strategies have been developed for fault diagnosis and fault-tolerant control in DC–DC converters, particularly in applications related to PV systems and renewable energy sources. These techniques primarily focus on detecting faults in power switches, such as IGBTs, as well as in passive components like diodes. The following section provides an overview of different approaches reported in the literature, addressing fault detection in DC–DC converters. These methods include sensor-based techniques, digital model-driven approaches, and implementations using reconfigurable platforms such as FPGAs. Each work presents varying degrees of complexity, detection accuracy, hardware requirements, and suitability for real-time applications. In [9], a hybrid approach is proposed for current balancing and open-circuit fault diagnosis in the IGBTs of interleaved boost converters. The method relies on monitoring diode and input current signals for fault detection, requiring current sensors at both points. This approach is particularly suitable for interleaved boost converters, which are commonly used in renewable energy applications. Similarly, [10] introduces a fault-tolerant strategy for PV DC–DC converters. The focus is on detecting open-circuit and short-circuit faults in the IGBTs through the analysis of boost control signals along with current and voltage sensors. This strategy is applied in PV systems with battery storage, aiming to enhance reliability and minimize system downtime caused by IGBT failures. In [11], detection and compensation schemes for open-circuit IGBT faults are presented for a modified boost DC–DC converter used in alternative energy systems. A fault detection index is introduced to accurately identify open-circuit conditions in the IGBT. The work presented in [12] proposes an FPGA-based real-time diagnostic method for IGBT faults in PV systems, coupled with a fault-tolerant DC–DC converter. The approach uses both current and voltage signals to identify open-circuit and short-circuit faults in the IGBT. A fault diagnosis and fault-tolerant control method for non-isolated DC–DC converters is described in [13]. This method is based on monitoring the inductor current as well as the input and output voltages of the converter, enabling effective fault detection and mitigation. In [14], a data-driven method is proposed for fault diagnosis in the switches of boost DC–DC converters used in PV applications. It uses existing current and voltage signals from the PV system, eliminating the need for additional sensors. This approach improves fault detection capabilities and system robustness, facilitating easier maintenance and uninterrupted operation of PV-based DC–DC converters. A DT-based fault diagnosis approach is presented in [15] for distributed PV systems. This method utilizes DT estimations and multiple system output signals to detect faults in PV panels, power converters, and electrical sensors. The method described in [16] focuses on diagnosing both open-circuit and short-circuit IGBT faults in non-isolated DC–DC converters using an FPGA. Fault detection is based on inductor current measurement and employs a hybrid architecture with two diagnostic subsystems to improve accuracy and responsiveness. In [17], a fault diagnosis and post-fault reconfiguration scheme is proposed for interleaved boost converters in PV energy systems. The fault detection relies on three indicators: (i) input current, (ii) switching frequency, and (iii) phase angle, to identify open-circuit faults in the IGBTs. Finally, [18] presents a technique for detecting both open-circuit faults (OCFs) and short-circuit faults (SCFs) in the diode and IGBT of the converter. The method uses diode voltage and the IGBT control signal to identify each fault type. However, since the converter’s input conditions are isolated, the method does not consider variations in input voltage during the fault detection process.

As shown in Table 1, most of the reviewed works primarily focus on fault detection in the IGBT. Only a few of them also address fault detection in the converter’s diodes. Furthermore, the generalizability of these methods is often limited. Another critical aspect is the input conditions applied to the converter in each study. In PV solar energy systems, the converter’s input varies due to changes in irradiance and panel temperature. Fast fluctuations in environmental conditions can lead to false positives in fault detection. For instance, a sudden drop in irradiance might be misinterpreted as an open-circuit fault in the power components, highlighting the importance of accounting for such variations. Additionally, several existing approaches assume isolated and idealized operating conditions, with limited validation under variable or real-world inputs. To address these limitations, this work proposes a generalized diagnostic method based on current and voltage measurements at both the input and output of a non-isolated power converter operating under variable input conditions. The proposed approach enables the detection of OCFs and SCFs in both the IGBT and the diode, as well as false data injection attacks (FDIAs) targeting the sensors used for converter control. A distinctive feature of this work is the integration of a hybrid diagnostic framework that combines a physics-based DT with data-driven classification using machine learning. This hybrid approach is still scarcely explored in the literature, despite its strong potential to improve fault detection accuracy, robustness, and interpretability. By using both physical modeling and learned patterns from operational data, the system can detect and classify anomalies while remaining generalizable to different operating conditions and PV plants configurations.

Therefore, the main contributions of this work are as follows:

1. Hybrid Digital Twin-Based Fault Diagnosis Framework: This work proposes a complete system that combines FMI-based [19] emulation (FMU), real-time data acquisition, feature extraction, and machine learning-based classification. This hybrid approach enables both fault detection and identification (diagnosis) in DC–DC converters within PV systems.
2. Detection of Multiple Fault Types: The proposed method effectively identifies not only classical hardware faults such as IGBT and diode open/short circuits but also false data injection attacks (FDIAs) targeting voltage and current sensors. This extends fault coverage to cyber-physical threats.
3. Scalable, Portable, and Low-Cost Architecture for Real-World Deployment: The proposed framework is designed with practical deployment in mind. Thanks to a physics-based normalization strategy, it maintains high performance across PV plants with different power ratings without requiring model retraining. Furthermore, the solution is fully compatible with low-cost embedded hardware such as Raspberry Pi 5 [20] and employs the FMI standard to ensure modularity and interoperability across different environments. These features make the system both scalable and portable, suitable for real-time operation in heterogeneous PV plants.

## 3. Photovoltaic Plant Digital Twin

**Figure 1:**
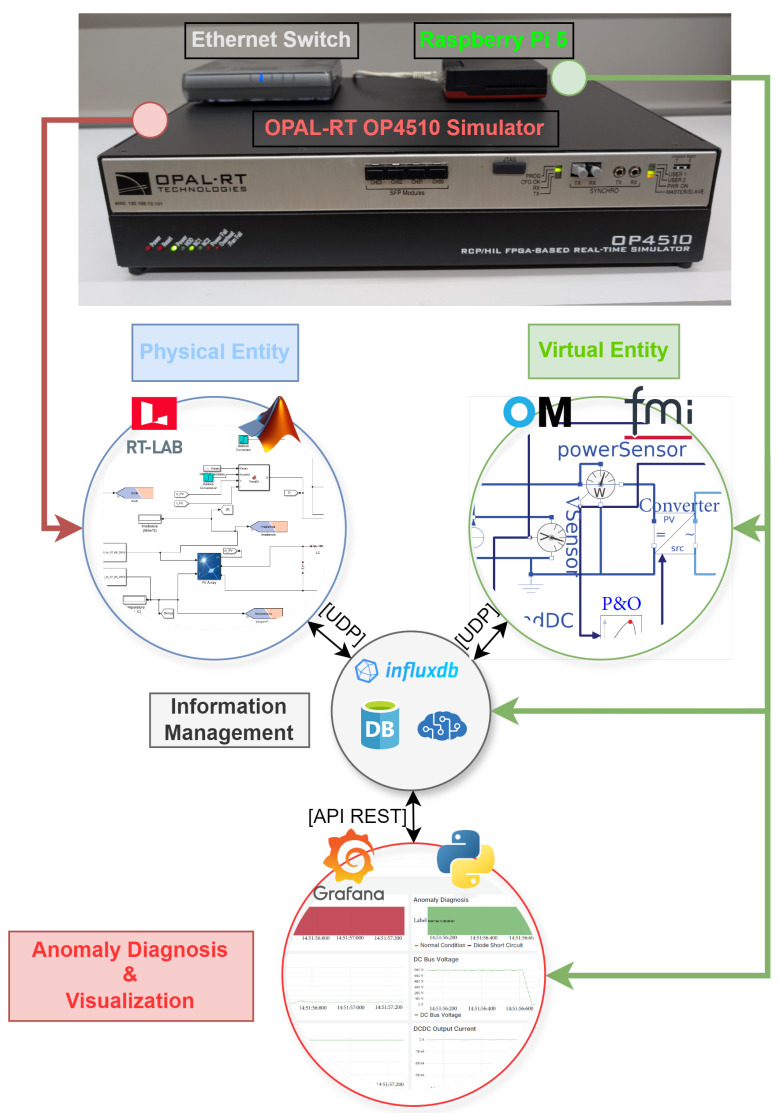
Five-dimensional digital twin architecture.

This section describes the development of the DT framework for fault diagnosis in a PV plant environment. The structure of the proposed DT is illustrated in Figure 1. A five-dimensional DT has been implemented, consisting of the following components: (i) Physical Entity; (ii) Virtual Entity; (iii) Information Management; (iv) Communications and (v) Services.

### 3.1. Physical Entity

**Figure 2:**
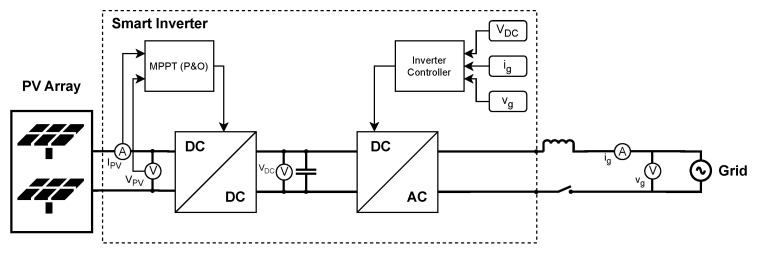
PV plant detailed model: PV array with a two-stage converter: (i) DC–DC boost converter with a maximum power point tracking (MPPT) controller based on the perturb and observe (P&O) method, and (ii) inverter with its controller; connected to the grid.

The physical entity has been implemented using OPAL-RT, enabling real-time hardware emulation. A detailed model of a PV plant (Figure 2) was developed and validated against real operational data from a real PV plant [21] to ensure accuracy and representativeness. OPAL-RT OP4510, Canada [22] was used to perform real-time emulation of the PV plant, with a time step of 10 ms, allowing for high-fidelity replication of dynamic system behaviour.

The following system parameters presented in Table 2 are used in order to validate the model against the real PV plant data of [21].

**Figure 3:**
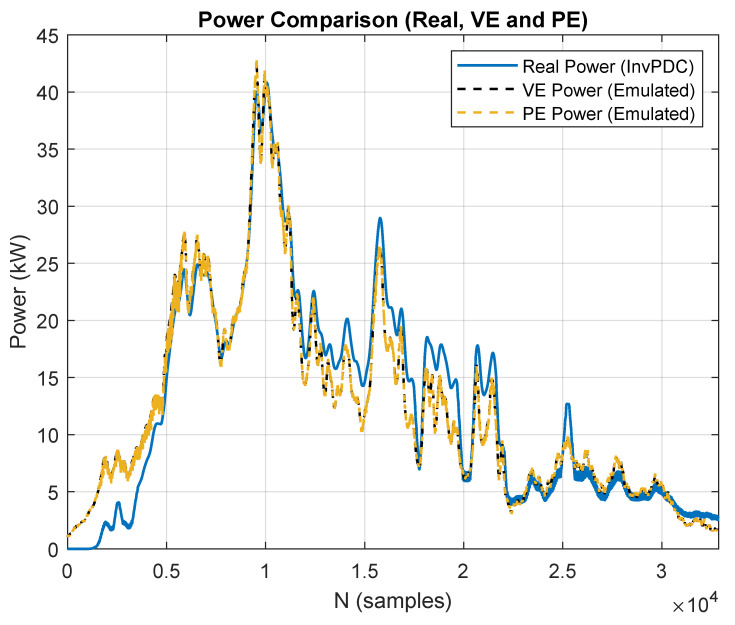
Virtual entity and physical entity validation (low irradiance scenario). Blue line (real power), black dashed line (virtual entity power), yellow dashed line (physical entity power).

Several validation tests were carried out to benchmark the model against real plant data. Figure 3 illustrates one of the most challenging days in the test set, a day with low irradiance and scattered clouds. The real power output is shown with a blue line, the physical entity (PE) with a yellow dashed line, and the virtual entity (VE) with a black dashed line. To quantify the model performance, the main error metrics are summarized in Table 3.

Even on this difficult day, the PE achieves a normalized-root-mean-square error (NRMSE) of 5.93 % with respect to the measured power. The slightly higher error stems from the plant having only a single irradiance sensor located at one point; on large-area PV plants, spatially variable irradiance on cloudy days can lead to local mismatches between the sensor reading and the irradiance actually seen by different strings. Nevertheless, the overall error remains low, confirming the robustness of the physical entity model.

Validating fault detection methods in PV systems under real fault conditions remains a major challenge, as publicly available datasets rarely contain labelled fault events. It is difficult to expect access to such data, since this would require long observation periods until faults naturally occur, and intentionally stopping a functioning plant to induce or record faults is not feasible, as it would lead to significant financial losses for power generation facilities. Consequently, the proposed approach has been validated primarily under normal operating conditions. Although operational data were available, they rarely contained documented fault events, which poses a significant challenge for performing supervised validation under faulty conditions. To address this limitation, an HIL architecture was employed. The physical platform used for real-time emulation is based on an OPAL-RT target executing a detailed Simulink model of the PV plant. This model incorporates official libraries from MATLAB Simulink 2020b and OPAL-RT, which have been previously validated against experimental setups [23]. The HIL configuration enables the injection of synthetic yet realistic fault scenarios with high temporal resolution, ensuring accurate representation of system dynamics during abnormal conditions (e.g., open circuit or short circuit faults). This approach not only enables repeatability and fine control over fault conditions but also aligns with recent trends in the literature [24,25], where HIL platforms such as OPAL-RT are increasingly adopted for validating PV system controllers and emulating realistic fault scenarios. Furthermore, it facilitates the generation of labelled synthetic datasets, which are instrumental in developing AI-based tools to enhance the intelligence and resilience of these systems.

### 3.2. Virtual Entity

The virtual entity of the DT has been designed with interoperability in mind, allowing integration across different industrial environments. To achieve this, a combination of OpenModelica v1.25.0 and the FMI standard framework has been employed.

#### 3.2.1. OpenModelica

OpenModelica has been used to design an averaged behavioural model of the PV plant. OpenModelica [26] is an open-source environment for the dynamic modeling and simulation of multi physical systems. It is based on the Modelica language, which enables the construction of complex, component-based system models. Specifically, the OpenModelica Connection Editor (OMEdit) was employed to develop a behavioural model of the PV system using the single diode model (SDM), a widely accepted representation of PV panel characteristics. The PV array is connected to an inverter system consisting of a boost converter stage followed by the inverter stage for grid connection. This virtual model replicates the behavior of the physical system in an averaged form, significantly reducing computational complexity. As a result, the model is well suited for deployment on resource-constrained embedded platforms. By adopting a lightweight yet representative DT, the system achieves a balance between execution accuracy and real-time performance, a key requirement for DT applications operating at the edge or in environments with limited computational resources.

The SDM includes a photocurrent source $I_{PH}$, proportional to solar irradiance, and a diode current $I_{d}$ described by the Shockley equation (see Equation (2)). The series resistance $R_{s}$ accounts for contact and material resistances within the cell, while the shunt resistance $R_{sh}$ models leakage currents and depends on the cell’s manufacturing process [27]. The output current and voltage of the panel are denoted as $I_{PV}$ and $V_{PV}$, as shown in Equations (1) and (2).

The implicit equation resulting from the electrical circuit of the SDM is as follows:

(1) $$I_{PV}=I_{PH}-I_{d}-\frac{V_{PV}+R_{s}\cdot I_{PV}}{R_{sh}}$$

The current $I_{d}$ is represented by the following equation:

(2) $$I_{d}=I_{SAT}\cdot \left(\exp\left(\frac{V_{PV}+R_{s}\cdot I_{PV}}{V_{t}\cdot a}\right)-1\right)$$

where $I_{SAT}$ is the reverse saturation current of the diode, *a* is the diode ideality factor, and $V_{t}$ is the thermal voltage of the diode, which is expressed as:

(3) $$V_{t}=\frac{K\cdot T}{q}$$

where *K* is the Boltzmann constant, *T* is the PV panel temperature in Kelvin, and *q* is the electron charge.

#### 3.2.2. FMI Standard Framework

The FMI is a standard designed to facilitate the exchange of models between different platforms and to enable platform-independent simulations [28]. The primary goal of the FMI standard is to streamline the development, storage, exchange, and reuse of dynamic system models across a wide range of simulation environments. This includes support for model-in-the-loop (MiL), software-in-the-loop (SiL), and HIL simulations, as well as applications in cyber-physical systems [19].

**Figure 4:**
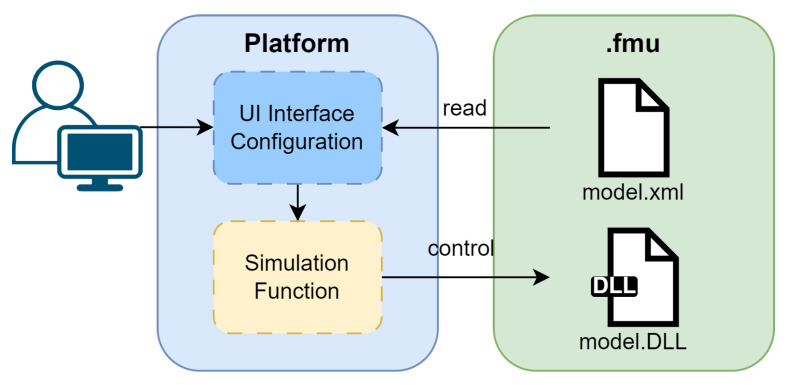
FMU functionality.

The use of FMUs provides several advantages in terms of modularity, portability, and interoperability. Figure 4 illustrates a typical interaction with a platform using an FMU. On the left, a user interacts with the configuration interface (blue), adjusting parameters relevant to the execution. These settings influence the control block (yellow), which is responsible for executing the appropriate control logic. On the right side, the FMU itself is shown. It consists of two main components: (i) model.xml, which describes the model structure, parameters, and metadata; and (ii) model.dll, which contains the compiled model logic and functions in C, specific to a given operating system. This architecture enables the efficient configuration and execution of models using encapsulated FMU files, promoting compatibility and reuse across heterogeneous frameworks.

A notable feature of FMUs is their ability to export model information. This enables the creation of “black-box” models, allowing developers to conceal internal details for intellectual property protection during commercialization. FMUs can then be deployed in any FMI-compliant environment such as Python, MATLAB, OpenModelica, or Ansys, facilitating the integration of components developed by engineers from different disciplines and promoting effective cross-domain collaboration.

#### 3.2.3. Virtual Entity Validation

The virtual entity has been validated following the same procedure used for the physical entity, as shown in Figure 3. The dynamic behavior of the virtual entity closely matches that of the physical entity. Additionally, Table 3 summarizes the error metrics between the physical and virtual entities. This validation is particularly relevant, as the physical entity is used in this work to emulate a photovoltaic plant under both normal operating conditions and fault scenarios.

### 3.3. Information Management

This subsection describes the complete data handling pipeline, including a structured storage in the database for subsequent use by the anomaly diagnosis service. The system is designed to operate in real time, ensuring that measurements from the physical system are reliably captured, filtered, and normalized before being inserted into the time-series database. The data flow begins with the reception of communication packets containing sensor readings from the PV plant, which are parsed and queued for processing. These signals are then used to compute diagnostic features and feed the DT-based fault detection mechanism. Simultaneously, all raw and processed signals are timestamped and logged into an InfluxDB [29] instance. This architecture allows for scalable operation of the anomaly detection pipeline and enables both real-time monitoring and historical analysis of PV plant behavior. All raw and processed signals, along with detection flags and classification outputs, are timestamped and stored in an InfluxDB time-series database. To support real-time monitoring and intuitive visualization, the system integrates Grafana [30], which provides interactive dashboards for inspecting signals, detecting anomalies, and tracking fault classifications in a user-friendly interface.

### 3.4. Communications

The DT communication is implemented via Ethernet using a custom communication protocol built on top of UDP. Data exchange occurs at a frequency of 10 ms, ensuring that the signals to be classified have enough resolution for the classifier to operate effectively. This communication framework interconnects the different dimensions of the DT, including the virtual entity, physical entity, information management, and services, enabling synchronized operation and data consistency across all components.

### 3.5. Services

The services provided by the DT represent its core functional objectives. For instance, one service is data visualization, through which the DT displays information and results generated by other components of the system, for example, with Grafana. Another key service is anomaly diagnosis, aimed at detecting faults and subsequently classifying them in two steps to support decision-making processes. Additionally, the DT can offer a synthetic data generation service, particularly valuable in industrial environments where access to real data is limited. In such scenarios, one of the main goals is to compensate for this limitation by generating artificial datasets, enabling the development and training of data-driven models.

## 4. Digital Twin Implementation

**Figure 5:**
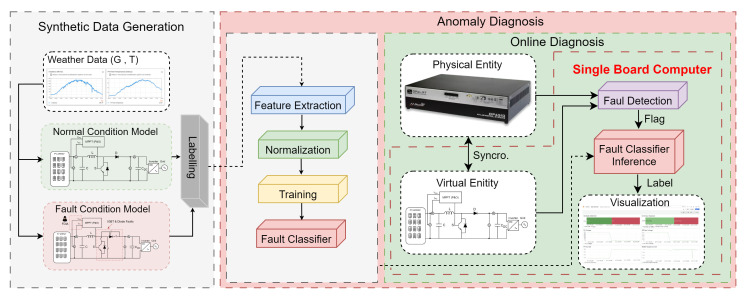
Digital twin implementation framework.

This section details the implementation of the DT, as illustrated in Figure 5, which outlines the implementation process of the DT. The implementation begins with a detailed physical model of the PV plant, emulated in real time using an HIL setup. This configuration enables the generation of synthetic data, which is automatically labelled by a dedicated process. The labelled dataset is then used to perform offline training of an anomaly classification model. Once trained, the model is deployed on an SBC, specifically, a Raspberry Pi 5 [20], to perform real-time fault diagnosis within the PV system.

### 4.1. Synthetic Data Generation Service

**Figure 6:**
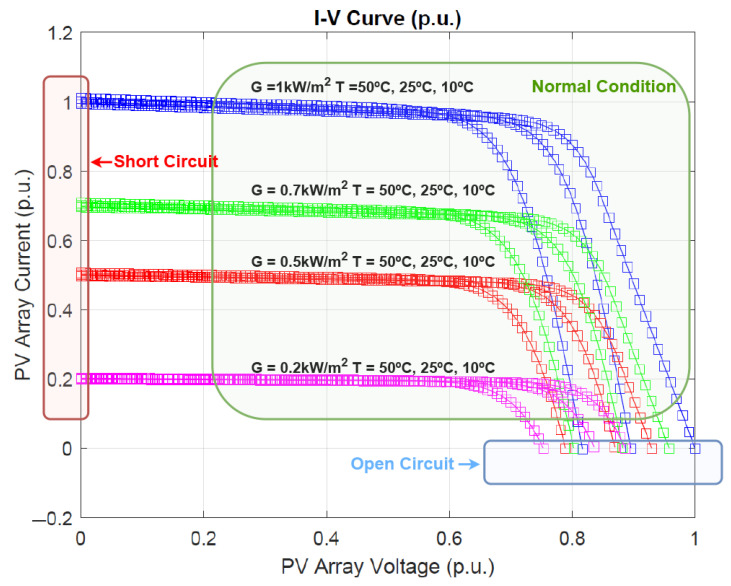
I–V curves of the PV technology under varying irradiance and temperature conditions, with the different operating regions marked.

Synthetic data generation was carried out with consideration of PV technology characteristics, as illustrated in Figure 6. Data were generated under varying irradiance and temperature conditions to comprehensively cover all possible operating modes of the system. It is important to note that the system’s dynamic behavior in response to a fault tends to shift toward specific regions of the I–V curve depending on the fault type. For instance, in the case of an open-circuit fault, the voltage approaches the open-circuit voltage while the current drops to zero. Conversely, in a short-circuit fault, the voltage falls to zero and the current approaches the short-circuit current. All other points along the I–V curve are considered to represent normal operating conditions. This fault-informed modeling ensures that the synthetic dataset realistically captures the system’s response to different failure scenarios, thereby improving the robustness and reliability of the diagnostic method.

Synthetic data were generated using two models: one representing normal operating conditions and another emulating fault scenarios. All tests were performed with a time step of 1 microsecond over a total duration of 1 s.

1. Windowing: A time window was extracted to capture the dynamic behavior of the fault in the sample range of 250,000:650,000, corresponding to a duration of 0.4s.
2. Resampling: A decimation factor of R=10,000 was applied to reduce the original sequence of m=400,000 samples to:
  (4) $$L_{\mathrm{resampled}}=\frac{650,000-250,000}{10,000}≃40\;\mathrm{samples},$$
  resulting in a compact matrix of dimensions 4 × 40, representing the four signal channels.
3. Labelling: The labelling process was guided by Table 4, which defines the fault categories and their associated class labels. Given the common limitation of data availability in industrial PV environments, this approach was designed to maximize the diagnostic utility of the available samples, ensuring that each instance contributes effectively to the training and evaluation of the anomaly diagnosis system.

### 4.2. Anomaly Diagnosis Service

The framework used in this work is illustrated in Figure 5 and comprises two main stages: (i) Offline Training, and (ii) Online Diagnosis. The Offline Training stage includes two key components. The first is responsible for the automatic generation and labelling of synthetic data. The second component performs feature extraction to identify the most relevant characteristics, with the goal of training a final classifier. In the Online Diagnosis stage, both the anomaly detector and classifier are deployed to evaluate their performance in detecting and classifying cyber-physical anomalies. These anomalies are identified in data originating from the physical entity of a PV plant emulated using HIL techniques.

#### 4.2.1. Offline Training

1. Feature Extraction: Raw signal windows are transformed into structured representations through a designed feature extraction process. Each observation window, comprising multichannel time series data, is mapped to a feature vector consisting of different numerical descriptors. These features aim to capture relevant statistical, temporal, and spectral characteristics from each signal channel.
  Table 5 summarizes the feature groups extracted from the multichannel signal window. Each group captures a different aspect of the signal: basic statistical moments, spectral properties via FFT, time-frequency information using wavelet decomposition (db4, level 2), and dynamic behaviours of the DC bus voltage. These features are concatenated into a single vector and used as input for the fault classification model. The selected features in Table 5 were chosen based on their relevance to the electrical behavior of PV systems and their ability to capture different aspects of the signal. First-order statistics provide general descriptive metrics, spectral features obtained via FFT identify frequency-domain anomalies, wavelet-based descriptors capture localized transient phenomena, and features derived from the DC bus voltage reveal dynamics critical to fault identification. This combination ensures both high discriminative power and interpretability, avoiding overfitting by limiting feature redundancy.
  To reduce redundancy and improve generalisation, a two-stage feature selection strategy was applied.
  Stage 1: Low-variance filtering.
  A low-variance filter was used to eliminate nearly constant features that do not contribute discriminative information. Specifically, features with variance below a fixed threshold were removed:
  (5) $$\sigma^{2}<10^{-3}$$
  This threshold strikes a balance between filtering out noise and retaining meaningful variability in the data.
  Stage 2: Embedded feature selection via mean decrease in impurity (MDI).
  An embedded selector based on Mean Decrease in Impurity was employed using a random forest with 300 trees. Each feature $f_{i}$ was assigned an importance score $I(f_{i})$, based on its contribution to impurity reduction across the forest. The subset of features was selected such that the cumulative importance satisfied:
  (6) $$\sum_{i=1}^{k}I\left(f_{i}\right)\geq 0.95\sum_{j=1}^{n}I\left(f_{j}\right)$$
  where *n* is the total number of features and k<n is the number of selected features accounting for 95% of the total importance.
  Both steps were executed independently within each fold of the nested cross-validation procedure to avoid information leakage and overfitting.
  This approach consistently reduced the dimensionality of the feature space by approximately 60%, without degrading the macro-F1 performance, and yielded a compact, interpretable feature set that preserved the most relevant descriptors for fault classification.
2. Normalization: Each channel signal $x_{c}\left(n\right)$ is normalized by its nominal value $k_{c}$, which corresponds to a characteristic voltage or current level. This normalization strategy ensures compatibility and portability across PV plants with different power ratings, yielding input signals within a comparable range regardless of system scale:
  (7) $${\tilde{x}}_{c}\left(n\right)=\frac{x_{c}\left(n\right)}{k_{c}},\;c\in \left\{V_{\mathrm{OC}},I_{\mathrm{SC}},V_{\mathrm{DC}},I_{\mathrm{SC}}\right\}.$$
  In practice, the normalization constants $k_{c}$ are selected based on standard operating conditions: (i) $I_{\mathrm{PV}}$ is normalized with respect to the short-circuit current (Isc) at 10 °C and 1000 W/m^2^ irradiance; (ii) $V_{\mathrm{PV}}$ is normalized with the open-circuit voltage (Voc) under the same standard conditions; (iii) $V_{\mathrm{DC}}$ is normalized using the nominal DC bus voltage plus a 10% margin to account for expected variations under normal operating conditions; and (iv) $I_{\mathrm{DC}}$ is normalized similarly to $I_{\mathrm{PV}}$, as both currents typically have similar magnitudes.
  This normalization process, applied during the preprocessing stage, improves the robustness and generalization capability of the diagnostic method across different systems and environmental conditions.
3. Fault Classifier Training: Table 6 presents the cross-validation performance (macro-averaged F1-score) and training times for the evaluated classification models. The goal of this comparison is to assess both the accuracy and computational efficiency of different algorithms in identifying fault types based on the extracted feature vectors. The selected hyperparameters for each model were chosen based on standard practices and preliminary tuning aimed at achieving a balance between performance and generalization. For ensemble-based methods such as Random Forest and Extra Trees, a moderately high number of estimators is used to ensure stability and low variance without incurring excessive computational cost. In gradient boosting models like XGBoost, typical configurations such as learning rate η=0.1 and tree depths of 3 to 6 were adopted to control model complexity and avoid overfitting. For the support vector machine (SVM) with RBF kernel, a regularization parameter of C=10 provided a good margin–flexibility trade-off, especially considering the high-dimensional feature space (134 descriptors). In the case of K-Nearest Neighbors, a small value of k=5 allowed the model to remain sensitive to local patterns while maintaining robustness. Among all models, Random Forest achieved the best performance, with an F1-macro score of 0.992, followed closely by XGBoost. The combination of extracted features and selected hyperparameters proved effective across multiple algorithmic families, highlighting the robustness of the proposed representation and its compatibility with a wide range of classifiers.
4. Physics informed rules: To enhance the reliability and interpretability of the anomaly classification process, a set of deterministic, physics-informed rules is applied after the machine learning (ML) stage. These rules are specifically designed to handle cases where the ML model may produce ambiguous or conflicting fault labels. By using physical domain knowledge and signal characteristics, these rules help refine and guide the final classification decision. A key challenge lies in distinguishing between an open-circuit fault in the IGBT and one in the diode, as their waveform signatures are very similar. By incorporating this domain-specific knowledge into the learning process, the classification performance can be significantly improved.
  Table 7 shows two representative examples. In the first case, when the classifier simultaneously suggests an open-circuit fault in both the IGBT and the diode, the decision is made based on the comparison of voltage deltas before and after the transient. If the upward voltage jump ($\Delta^{+}$) is greater than the downward jump ($\Delta^{-}$), the fault is attributed to an open IGBT. In the second case, when there is ambiguity between a current or voltage bias, the mean deviation from the nominal values is computed for both signals. The fault is attributed to the signal exhibiting the greater deviation, thereby indicating whether the anomaly is more consistent with a voltage or current bias. This rule-based postprocessing layer serves as a lightweight and interpretable mechanism to enhance diagnostic robustness and reduce false positives.

#### 4.2.2. Online Diagnosis

1. Data Acquisition and Storage:
  UDP packets are received every 1 ms, each containing eight double-precision values: PV plant power, emulation clock, irradiance, temperature, PV array voltage, PV array current, DC bus voltage, and DC–DC output current. From these, the last four measurements: PV array voltage, PV array current, DC bus voltage, and DC–DC output current, are used to construct a time-windowed observation vector for subsequent analysis and fault diagnosis.
  (8) $$x_{i}=\left[\begin{array}{c} \mathrm{PV}\;\mathrm{Array}\;\mathrm{Voltage}\\ \mathrm{PV}\;\mathrm{Array}\;\mathrm{Current}\\ \mathrm{DC}\;\mathrm{Bus}\;\mathrm{Voltage}\\ \mathrm{DCDC}\;\mathrm{Output}\;\mathrm{Current} \end{array}\right]$$
  Each of these four signal channels is stored in a circular buffer of fixed length L=40, defined as:
  (9) $${\mathrm{Buffer}}_{j}=\left\{x_{j,t-L+1},\ldots ,x_{j,t}\right\},\;j\in \left\{1,2,3,4\right\}$$
2. Fault Detection: Fault detection is carried out using the virtual entity developed in the previous section. The detection mechanism relies on the comparison between emulated and real measurements from the physical system. When a significant discrepancy is observed i.e., a sudden deviation in the real power, current, or voltage compared to the virtual entity estimation, an alert flag is triggered. To determine whether or not a discrepancy is significant, a threshold is defined based on the results of prior validation tests. If the deviation between virtual and real values exceeds this threshold, the system considers that an anomaly or fault has occurred, activates the corresponding flag, and launches the classifier to identify the fault type. Data from the physical entity are received at a fixed timestep of Δt=0.01s. At each step, the simulated power $P_{\mathrm{virtual}}$, current $I_{\mathrm{virtual}}$, and voltage $V_{\mathrm{virtual}}$ are computed by the FMU and compared with the real measurements from the HIL physical entity: $P_{\mathrm{real}},\;I_{\mathrm{real}},\;V_{\mathrm{real}}$.
  The absolute power error is calculated as:
  (10) $$\Delta P=|P_{\mathrm{virtual}}-P_{\mathrm{real}}|$$
  And the relative errors in voltage and current are defined as:
  (11) $$\begin{array}{cc} \epsilon_{V} & =\left|\frac{V_{\mathrm{virtual}}-V_{\mathrm{real}}}{V_{\mathrm{real}}+\varepsilon }\right|\\ \epsilon_{I} & =\left|\frac{I_{\mathrm{virtual}}-I_{\mathrm{real}}}{I_{\mathrm{real}}+\varepsilon }\right| \end{array}$$
  where $\varepsilon =10^{-6}$ is a small constant added to avoid division by zero.
  (12) $$\Delta P>P_{\mathrm{threshold}}\;\mathrm{or}\;\epsilon_{V}>\epsilon_{V,\mathrm{threshold}}\;\mathrm{or}\;\epsilon_{I}>\epsilon_{I,\mathrm{threshold}}$$
  These thresholds have been defined based on the validation tests presented in Section 3, where it was observed that, even on one of the worst-performing days, the error between the virtual and the physical entities was only 0.61%. Therefore, the selection of the threshold can be adjusted depending on how conservative the desired detector is, always considering the validation error margins established during validation.
3. Fault Classification: For this stage of the anomaly diagnosis service, the classifier developed in the previous section is utilized. When the detector flag is triggered, the classifier retrieves four signal traces, each consisting of 40 samples, to identify the type of anomaly. Once the anomaly has been classified, the result is sent to the database for storage and subsequent representation through the visualization service.

### 4.3. Visualization Service

For the visualization service, Grafana was selected due to its strong compatibility with the InfluxDB time-series database. This compatibility allows for effective representation of the data stored in the database. The data transmitted to InfluxDB includes measurements from the physical entity and the virtual entity and the outputs of the anomaly detection and classification modules. As detailed in the following section, all relevant signals are visualized in Grafana, enabling the identification of the exact time an anomaly occurred, as well as the corresponding anomaly type.

### 4.4. DT Implementation and Cost Analysis

The proposed system has been implemented on low-cost hardware to ensure its accessibility and applicability in real-world industrial environments, particularly in PV installations. The chosen platform is the Raspberry Pi 5, a cost-effective and energy-efficient SBC that offers sufficient computational resources to host the virtual entity, manage the information infrastructure, and execute the anomaly diagnosis service of the DT presented in this work. The Raspberry Pi 5 features a multi-core processor and up to 8 GB of RAM, enabling the deployment of lightweight machine learning models and real-time data processing tasks without the need for cloud computing or expensive embedded platforms. Its compatibility with common Linux distributions and open-source software frameworks simplifies integration with industrial communication protocols and IoT platforms. This edge-oriented implementation strategy minimizes latency, reduces dependency on network connectivity, and enhances data privacy by keeping sensitive operational data within the local system. Furthermore, the affordability of the hardware (under USD 100) makes the solution scalable and attractive for widespread deployment across distributed energy resources.

## 5. Results

**Figure 7:**
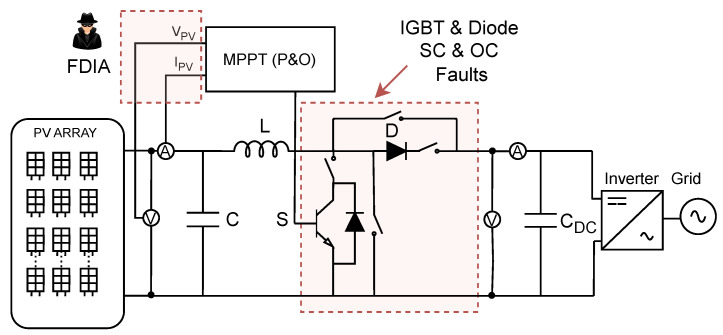
Photovoltaic plant architecture with IGBT and diode open- and short-circuit faults, and FDIAs on MPPT sensors.

This section analyzes the results obtained and evaluates the generalization capability of the developed model. The main goal is to assess whether or not the proposed approach is effective for diagnosing the anomalies represented in Figure 7. Additionally, the transferability of the system to other PV plants (see Table 8) whose power characteristics differ from those used during the synthetic data generation phase is examined. To this end, the algorithm was tested on a PV plant with nominal conditions that differ from the original setup. Thanks to the normalization process applied prior to classification, the only required adaptation involves updating the nominal values to match those of the new plant. This ensures that the input variables remain within the normalized range of [0, 1]. By doing so, the classifier is able to focus exclusively on the dynamic patterns of the time series, independently of the absolute scale of the signals. This enhances the robustness and portability of the system when deployed across varying plant configurations.

Tests have been carried out to evaluate the resulting classifier using the emulator, specifically 500 tests. The system achieved an overall accuracy of 0.946, indicating a good overall performance of the classifier architecture.

### 5.1. Metrics by Fault Type

**Figure 8:**
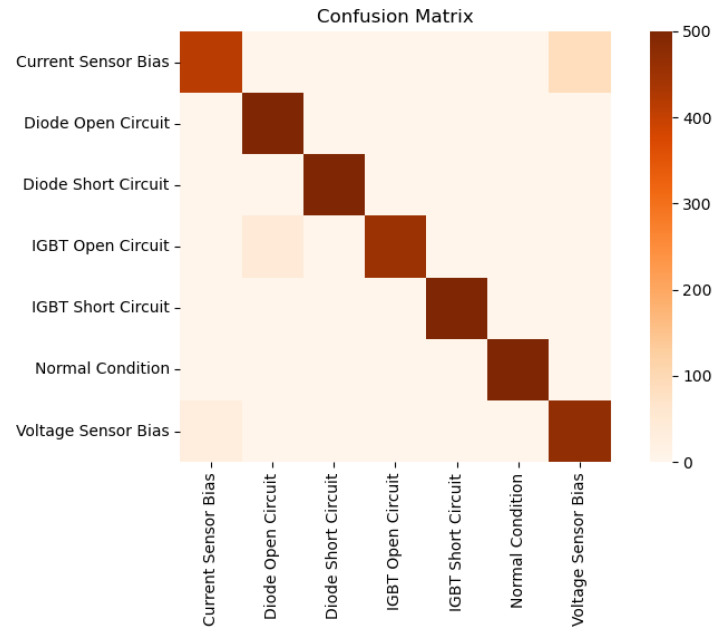
Confusion matrix of online diagnosis.

The results shown in Table 9 and Figure 8 demonstrate that the system is highly effective at detecting most fault conditions, particularly critical faults such as Diode Short Circuit and IGBT Short Circuit, for which perfect precision, recall, and F1-scores are achieved. It can also be observed that the system distinguishes well between Diode Open Circuit and IGBT Open Circuit, although there are a few misclassifications due to the similarity of the waveform patterns associated with these fault types.

The Voltage Sensor Bias condition shows the lowest precision (0.825), indicating a higher rate of false positives, mainly because it can be confused with other faults such as Current Sensor Bias. Similarly, Current Sensor Bias exhibits a moderately low recall (0.804), suggesting that some fault instances are not being correctly identified. This is likely because, when a bias occurs in either voltage or current, it often causes a cascading effect that also distorts the other variable, making the faults difficult to isolate.

To address these challenges, future work may explore the use of physics-informed machine learning (PIML) techniques [31], which integrate known physical relationships, such as current–voltage dependencies, into the training model structure. By constraining the learning process with system-level knowledge, PIML can help improve class separability, especially for faults with overlapping signatures, and enhance recall for difficult cases like the Current Sensor Bias.

Detection of the Normal Condition is nearly perfect, with an F1-score of 0.999, which is advantageous for minimizing false alarms in healthy system states.

**Figure 9:**
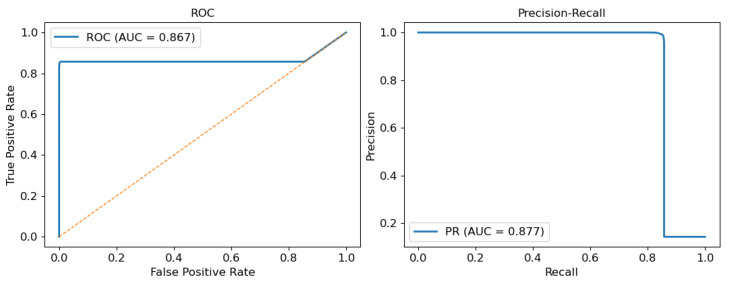
Model evaluation using ROC and Precision–Recall curves.

The performance of the model was evaluated using the ROC and Precision–Recall curves, as shown in Figure 9. The ROC curve demonstrates strong discriminative ability, with an area under the curve (AUC) of 0.867, indicating the model’s robustness in distinguishing between positive and negative classes.

The Precision–Recall curve yields an AUC of 0.877, which is particularly valuable in scenarios with class imbalance. This result shows that the model maintains high precision even as recall increases, meaning it correctly identifies a large proportion of true positives without significantly increasing the number of false positives.

Together, these metrics suggest that the model exhibits reliable behavior, achieving a favourable balance between sensitivity and precision.

### 5.2. IGBT Short Circuit Fault

**Figure 10:**
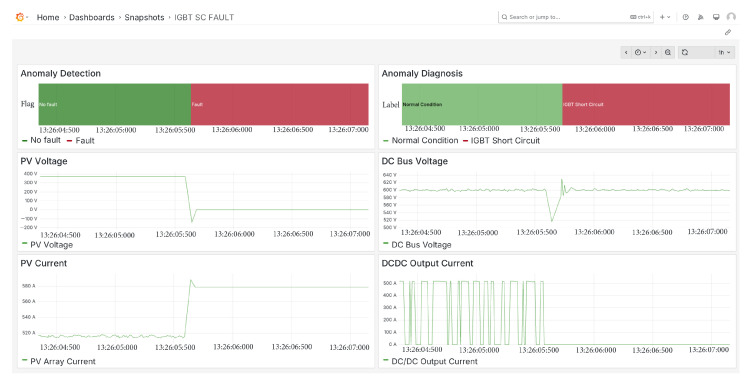
IGBT short circuit fault diagnosis.

The first fault introduced into the system is a short-circuit fault in the IGBT. As shown in Figure 10, the anomaly detector promptly raises a fault flag, and a few milliseconds later the classifier correctly identifies the fault as an IGBT short circuit. Additionally, it can be observed that the PV array voltage drops to zero, while the current rises to the short-circuit current level, consistent with the expected electrical behavior under this fault condition.

### 5.3. Diode Short Circuit Fault

**Figure 11:**
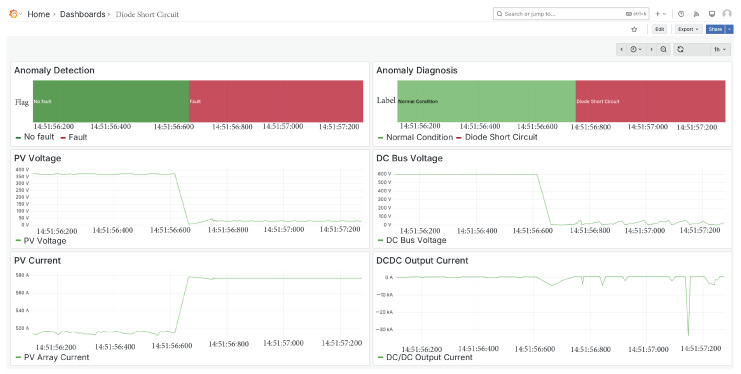
Diode short circuit fault.

The second fault introduced into the system is a short-circuit fault in the diode, as illustrated in Figure 11. It can be observed that the PV array voltage drops to zero, while the current approaches the short-circuit current level. Additionally, the DC bus voltage also tends toward zero, consistent with the expected system response under this fault condition.

In this case, the anomaly detector was triggered a few milliseconds after the fault occurred, subsequently activating the classifier, which correctly identified the fault as a diode short circuit.

### 5.4. IGBT Open Circuit Fault

**Figure 12:**
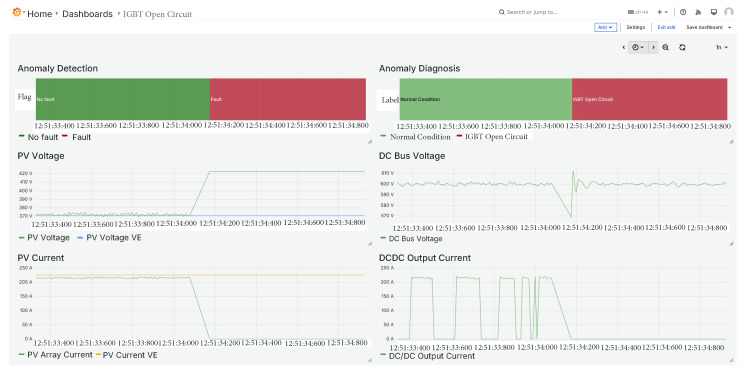
IGBT open circuit fault.

The third fault introduced is an open-circuit fault in the IGBT, as illustrated in Figure 12. In this scenario, the PV array voltage approaches the open-circuit voltage, while the current drops to zero, which is consistent with the expected behavior for this type of fault.

Additionally, both the DC bus voltage and the DC–DC output current exhibit distinct transient responses that play a critical role in enabling the classifier to accurately identify the fault as an IGBT open circuit.

### 5.5. Diode Open Circuit Fault

**Figure 13:**
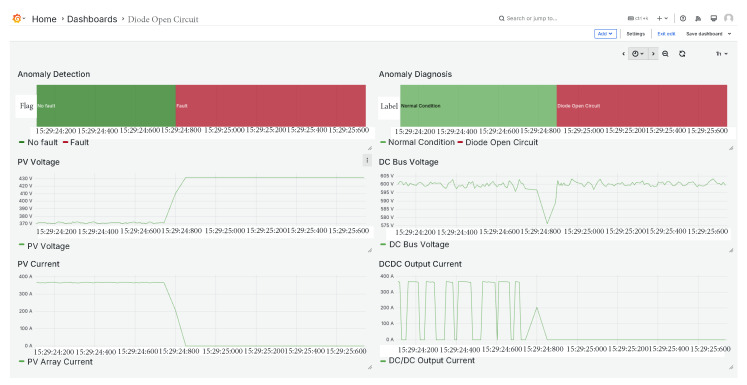
Diode open circuit fault.

The fourth fault introduced is a diode open-circuit fault, as shown in Figure 13. This type of fault exhibits a dynamic behavior very similar to the previously analysed IGBT open-circuit case. As observed, the PV array voltage tends toward the open-circuit voltage, while the current drops to zero.

However, the transient responses of the DC bus voltage and the DC–DC output current show subtle differences. These variations are effectively captured by the classifier, which successfully identified the fault as a diode open circuit.

### 5.6. PV Array Current Sensor FDIA

**Figure 14:**
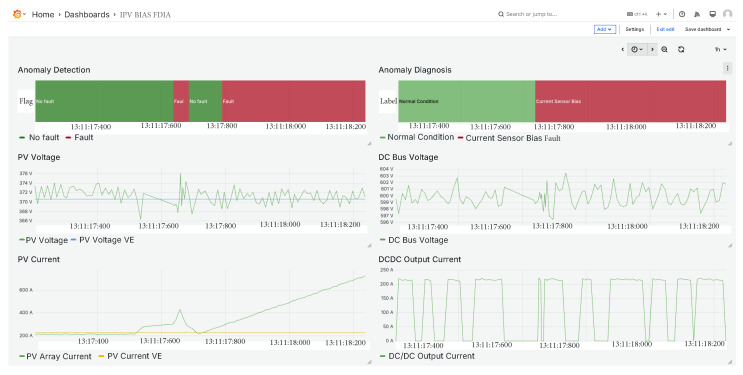
PV array current sensor FDIA.

The fifth fault corresponds to an FDIA targeting the current sensor of the PV array. As shown in Figure 14, the current signal is initially perturbed, then briefly returns to normal values, and subsequently deviates again toward anomalous behavior. Since the anomaly detector is threshold-based, it can be observed that the system temporarily returns to a normal condition when the manipulated values fall within acceptable limits. Despite these fluctuations, the classifier correctly identified the fault as a current sensor anomaly, demonstrating its robustness in detecting intermittent or stealthy FDIAs.

### 5.7. PV Array Voltage Sensor FDIA

**Figure 15:**
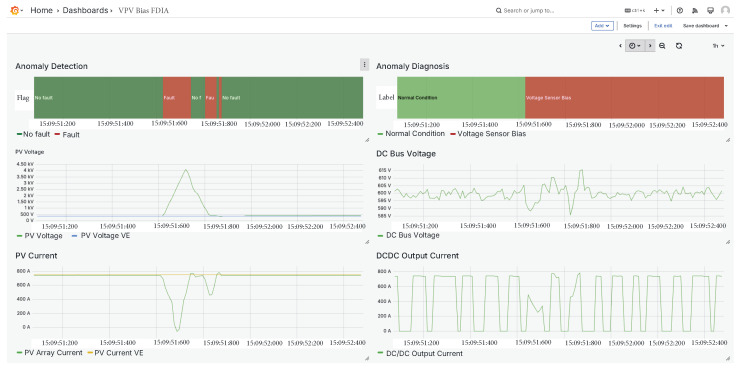
PV array voltage sensor FDIA.

Finally, an FDIA was tested on the voltage sensor, as illustrated in Figure 15. In this scenario, the voltage measurement was manipulated, which eventually disrupted the MPPT control algorithm. As a result, both the current and power output of the system were also affected. Despite the cascading impact on multiple signals, the classifier successfully detected the anomaly and correctly identified it as a voltage sensor FDIA, demonstrating its effectiveness even under complex fault conditions.

As demonstrated, the framework developed in this work has been successfully validated through real-time testing. The anomaly detection module reliably triggered alerts when the predefined thresholds—configured by the user—were exceeded. Once the classifier was invoked, it accurately identified the corresponding fault condition.

This real-time validation confirms both the responsiveness and correctness of the system under dynamic operating conditions. The detection thresholds proved effective in discriminating between normal behavior and abnormal patterns, while the classifier maintained high accuracy in assigning the correct fault labels based on extracted features. These results support the practical viability of the proposed framework for deployment in real-world PV systems.

### 5.8. Deployment Analysis on a Low-Cost Platform

**Figure 16:**
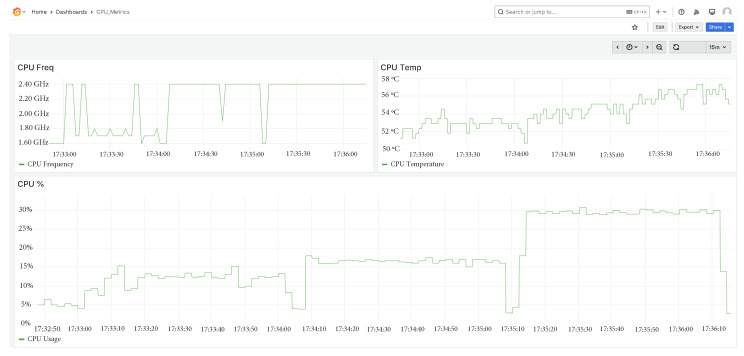
CPU metrics during the three-step stress test.

A three-step stress test was executed with 8, 96, and 192 channels sampled at 100 Hz, each load level lasting 60 s. The results—summarised in Figure 16—confirm that a Raspberry Pi 5 provides ample computational headroom for real-time execution:

- 8 channels: CPU utilisation stabilises at 10 %.
- 96 channels: the load rises modestly to 17 %.
- 192 channels: even at the highest load, the CPU peaks at only 30 %.

Throughout the experiment, the FMU-based virtual entity maintained an average execution time of 2.83 ms, leaving generous slack for the remaining tasks.

If future use-cases (e.g., additional sensor channels or new fault classifiers) push the Raspberry Pi 5 beyond its safe real-time envelope, three complementary strategies can extend the deployment:

(i) Horizontal scaling: When the channel count exceeds the real-time capacity of a single Raspberry Pi, extra boards can be added in a mesh and synchronised via NTP. Each node handles local acquisition and FMU-based diagnosis, whereas an MQTT broker [32] hosted on any node or a lightweight edge server will carry out the high-level diagnostics. The architecture scales linearly with the number of nodes while keeping per-device latency constant.
(ii) Hot-plug detectors: Diagnostic algorithms are containerised as micro-services that can be loaded or unloaded without stopping the main loop. Adding a new fault type therefore introduces only the incremental CPU cost of its container, leaving existing detectors untouched.
(iii) Adaptive off-loading: If sustained CPU utilisation exceeds 80%, the framework can switch to an off-load mode: (a) raw feature vectors are compressed and forwarded to the cloud, or (b) only anomaly flag detection is published, while detailed analysis is deferred to a more powerful backend.

This results shows that the approach presented in this work remains real-time capable on low-cost hardware while offering a clear migration path for larger or more complex deployments.

## 6. Conclusions

This work has introduced a comprehensive and transferable fault diagnosis framework for DC–DC converters in PV systems. By using a DT implemented via the FMI standard, the system enables accurate emulation of key electrical variables and facilitates real-time fault detection through continuous comparison with physical measurements.

The integration of statistical, spectral, and wavelet-based features with supervised classification models has proven effective in detecting a wide range of fault types. The proposed framework specifically targets both open circuit and short circuit faults in IGBTs and diodes, as well as FDIAs affecting voltage and current sensors. By addressing both hardware-level anomalies and sensor-layer cybersecurity threats, the system exhibits a high level of robustness and completeness. Unlike other methodologies in the literature, which frequently focus on a narrow subset of faults, this work stands out by incorporating a DT for fault detection, followed by machine learning-based classification.

The normalization strategy allows the system to operate reliably across PV plants with varying power ratings, avoiding the need for retraining when deployed in new environments. Real-time validation using OPAL-RT confirmed the system’s responsiveness and diagnostic accuracy under dynamically changing operating conditions. Moreover, its successful deployment on a low-cost embedded platform underscores its potential for widespread adoption in industrial PV monitoring applications.

Future work will focus on developing more accurate models and expanding the fault taxonomy to cover a broader range of components typically present in PV plants. This includes extending diagnosis capabilities to additional sensors, passive elements, and auxiliary systems beyond the DC–DC converter. Additionally, future work will focus on integrating fault-tolerant control structures to close the loop of the DT framework. This enhancement would enable not only the diagnosis of faults but also real-time corrective actions on the PV plant, thereby increasing system resilience and potentially preventing faults from escalating into failures.

## Figures and Tables

**Table 1:** Comparison of fault diagnosis methods for DC–DC converters.

Reference	Transferability	Required Signals	Fast Variable DC Input Source	Type of Faults	Topology	Digital Twin Approach
[9]	Limited	Diode current and input current	None	Switch OCF	Interleaved boost converter	No
[10]	Limited	Control signals of the converter, input and output current and voltage	Yes	Switch OCF and SCF	Three level boost converter	No
[11]	Limited	Current and voltage signals	None	Switch OCF	Modified boost converter	No
[12]	Limited	Voltage and current signals	None	Switch OCF and SCF	Single switch boost converter	No
[13]	Limited	Control signals, inductor current, input and output voltages	None	Switch OCF and SCF	Nonisolated boost converter	No
[14]	Limited	PV current and voltage	Yes	Switch OCF and SCF	Single switch boost converter	No
[15]	Yes	System output and DT estimation	None	Switches OCF and SCF, sensor faults	Four switch buck-boost converter	Yes
[16]	Limited	Inductor current slope	None	Switch OCF and SCF	Nonisolated single-ended boost converter	No
[17]	Limited	Input current, switching frequency component, phase angle	Yes	Switch OCF	Interleaved boost converter	No
[18]	Limited	Diode voltage and IGBT control signal	None	Switch and Diode OCF and SCF	Single switch buck converter	No
This work	Yes	Input and output current and voltage	Yes	Switch and diode OCF and SCF, FDIA sensors	Single switch boost converter	Yes

**Table 2:** PV plant parameters for physical entity emulation.

Parameter	Value
Array Rated DC Power [kW] ($P_{dc}$)	100
Module Model	SunPower SPR-305E-WHT-D, EE.UU
Module Rated Power [W]	305.226
Modules Per String	5
Number of Source Circuits	66
DC Bus Voltate [V]	500
Inverter Power [kW]	100

**Table 3:** Statistical comparison between real power experimental data and VE/PE models.

Comparison	NRMSE (%)	R^2^
VE vs. Real	6.05	0.93
PE vs. Real	5.92	0.93
VE vs. PE	0.61	0.99

**Table 4:** Fault labels of DC–DC faults.

Faulty Component	Label
No fault	Normal Condition
IGBT OC	Open Circuit IGBT
IGBT SC	Short Circuit IGBT
Diode OC	Open Circuit Diode
Diode SC	Short Circuit Diode
FDIA Voltage Sensor	Voltage Sensor Fault
FDIA Current Sensor	Current Sensor Fault

**Table 5:** Features taxonomy.

Group	Features	Key Formulas
1st-order Statistics	4 × 8	$\mu ;\sigma ;\mathrm{skew};\kappa ;\min;\max;\sum x^{2}$
FFT Spectrum	4 × 2	$H=-\sum p_{i}\log p_{i}$, $f_{c}=\sum f_{i}p_{i}$
Wavelets db4 (L=2)	4 × 6	μ,σ on A2, D2, and D1
DC Bus Dynamics	4	$\max\dot{V};\min\dot{V};V_{\mathrm{peak}}-V_{0};V_{0}-V_{\mathrm{valley}}$

**Table 6:** Cross-validation performance (macro F1-score) of the evaluated models.

Model	Key Parameters	F1 Score	Training Time [s]
Random Forest	$n_{\mathrm{tree}}=300$	0.992 ± 0.010	2
XGBoost	η=0.1, depth = 6	0.987 ± 0.022	4
SVM–RBF	C=10	0.973 ± 0.031	14
KNN	k=5	0.964 ± 0.035	<1
Extra Trees	300 trees	0.962 ± 0.029	2

**Table 7:** Physics informed rules applied after the ML stage.

Conflicting Case	Physical Metric	Decision
IGBT OC vs. Diode OC	$\Delta^{+}=V_{\mathrm{peak}}-V_{0}$, $\Delta^{-}=V_{0}-V_{\mathrm{valley}}$	OC $⇐\Delta^{+}>\Delta^{-}$
Current vs. Voltage Bias	$|{\bar{V}}_{\mathrm{PV}}-V_{0}|$ vs. $|{\bar{I}}_{\mathrm{PV}}-I_{0}|$	Voltage Bias ⇐ first is greater

**Table 8:** PV plant parameters for online diagnosis.

Parameter	Value
Array Rated DC Power [kW] ($P_{dc}$)	271
Module Model	Sharp NU-U235F2, Japan
Module Rated Power [W]	235
Modules Per String	12
Number of Source Circuits	96
DC Bus Voltate [V]	600
Inverter Power [kW]	260

**Table 9:** Summary of evaluation metrics by fault type.

Fault Type	Precision (P)	Recall (R)	F1-Score
Current Sensor Bias	0.918	0.804	0.857
Diode Open Circuit	0.901	1.000	0.948
Diode Short Circuit	1.000	1.000	1.000
IGBT Open Circuit	1.000	0.890	0.942
IGBT Short Circuit	1.000	1.000	1.000
Normal Condition	0.998	1.000	0.999
Voltage Sensor Bias	0.825	0.926	0.873

## Data Availability

Data are contained within the article.

## Abbreviations

The following abbreviations are used in this manuscript:

FMI	Functional Mock-Up Interface Standard
FMU	Functional Mock-Up Unit
FDIA	False Data Injection Attack
DT	Digital Twin
PV	Photovoltaic
SBC	Single Board Computer
MiL	Model-in-the-Loop
SiL	Software-in-the-Loop
HIL	Hardware-in-the-Loop
SDM	Single Diode Model
OMEdit	OpenModelica Connection Editor
SCF	Short Circuit Fault
OCF	Open Circuit Fault
